# Evaluation of Tyrosine Kinase Inhibitor Combinations for Glioblastoma Therapy

**DOI:** 10.1371/journal.pone.0044372

**Published:** 2012-10-02

**Authors:** Avadhut D. Joshi, Watcharin Loilome, I-Mei Siu, Betty Tyler, Gary L. Gallia, Gregory J. Riggins

**Affiliations:** Department of Neurosurgery, School of Medicine, Johns Hopkins University, Baltimore, Maryland, United States of America; The University of Chicago, United States of America

## Abstract

Glioblastoma multiforme (GBM) is the most common intracranial cancer but despite recent advances in therapy the overall survival remains about 20 months. Whole genome exon sequencing studies implicate mutations in the receptor tyrosine kinase pathways (RTK) for driving tumor growth in over 80% of GBMs. In spite of various RTKs being mutated or altered in the majority of GBMs, clinical studies have not been able to demonstrate efficacy of molecular targeted therapies using tyrosine kinase inhibitors in GBMs. Activation of multiple downstream signaling pathways has been implicated as a possible means by which inhibition of a single RTK has been ineffective in GBM. In this study, we sought a combination of approved drugs that would inhibit *in vitro* and *in vivo* growth of GBM oncospheres. A combination consisting of gefitinib and sunitinib acted synergistically in inhibiting growth of GBM oncospheres *in vitro*. Sunitinib was the only RTK inhibitor that could induce apoptosis in GBM cells. However, the *in vivo* efficacy testing of the gefitinib and sunitinib combination in an EGFR amplified/ PTEN wild type GBM xenograft model revealed that gefitinib alone could significantly improve survival in animals whereas sunitinib did not show any survival benefit. Subsequent testing of the same drug combination in a different syngeneic glioma model that lacked EGFR amplification but was more susceptible to sunitinib *in vitro* demonstrated no survival benefit when treated with gefitinib or sunitinib or the gefitinib and sunitinib combination. Although a modest survival benefit was obtained in one of two animal models with EGFR amplification due to gefitinib alone, the addition of sunitinib, to test our best *in vitro* combination therapy, did not translate to any additional in vivo benefit. Improved targeted therapies, with drug properties favorable to intracranial tumors, are likely required to form effective drug combinations for GBM.

## Introduction

Improving therapy for patients with Glioblastoma multiforme (GBM) is one of the biggest challenges in oncology. Although molecular targeting has shown success in many cancers, targeted therapy for GBM has yet to demonstrate an appreciable clinical survival benefit [1], [2]. For example, targeting of Epidermal Growth Factor Receptor (EGFR) with small molecules or monoclonal antibodies has been reported to offer no survival benefit [1], despite the fact that EGFR is the most common genomically altered oncogene in GBM, and targeting EGFR has shown benefit in other cancers. So an important question is: can targeted therapy provide a benefit to GBM patients?

The oncogenic receptor tyrosine kinases (RTKs) that are mutated in GBM are obvious molecular targets and many small molecule inhibitors of the RTKs are available. A mutation analysis of over 20,000 gene coding regions in GBM genomes confirmed that the RTK/PI3K/AKT pathway is one of the most frequently altered groups of genes in GBM [3]. The commonly altered genes include EGFR (40% approximate frequency), PTEN (37%), PIK3CA (13%), PIK3R1 (8%) and PDGFRA (8%) [3], [4]. Over 80% of glioblastomas have an acquired alteration in the RTK/PI3K/AKT pathway with about 40% of tumors having some alteration in EGFR [3], [5] suggesting that scarcity of a prevalent alteration is not the problem with targeted therapy in most GBMs. However, in spite of recent advances in development of targeted therapies, RTK inhibitors have shown negligible success against GBMs.

Lack of successful therapies against GBMs using RTK inhibitors raises several questions. Are the molecular targeting agents reaching and inhibiting the presumed target effectively in GBM? What are the resistance mechanisms involved if the inhibitors are reaching the tumor in effective concentrations? Growth signaling through alternate pathways, as well as tumor heterogeneity could be two of many factors involved in tumor resistance mechanisms.

In the following study, we tried to evaluate a series of RTK inhibitors in GBM systems *in vitro* and *in vivo* to determine if we could find a combination of RTK inhibitors that would be more successful than a single agent. The premise of the work was to evaluate approved inhibitors designed to target the most frequently activated tyrosine kinases in GBMs. The best *in vitro* pair of drugs inhibited GBM oncospheres synergistically was gefitinib and sunitinib. However, the improved activity of RTK combination did not perform as predicted *in vivo*. Gefitinib alone had a significant but modest survival benefit in a GBM xenograft mouse model mouse model. Moreover, *in vivo* evaluation of the same drugs in a syngeneic rat model of GBM failed to provide any survival benefit. Although the single agent therapy might show activity in certain genetic backgrounds, combinations that effectively target multiple RTK pathways in an intracranial target are needed.

## Results

### Glioblastoma Oncospheres Have Activation of Multiple Tyrosine Kinases

Our first goal was to develop *in vitro* cell-based assays for detecting activity of RTK inhibitors and combinations of inhibitors. For this we deemed it important that the cell lines were: 1) from human GBM patients 2) had relevant RTK pathway mutations or activation and 3) formed invasive grade IV astrocytomas when injected intracranially in nude mice.

Therefore, we employed GBM oncospheres for determining the effects of the RTK inhibitors on proliferation and cell death. Oncospheres, also referred to as stem-like cell cultures, grow in suspension using serum-free stem cell media. This culturing system appears to maintain genomic and phenotypic changes of the primary tumor better than traditional cell lines [6].

We used two GBM oncosphere lines for screening drug combinations. The 020913 GBM cell line maintains the primary tumor EGFR amplification as determined by a genomic copy number analysis [4]. EGFR amplification is normally lost in serum-based adherent cultures, but appears to be maintained by oncospheres, and found in over a third of primary GBM samples [4].

The 060919 GBM cell line was derived from a xenograft tumor that was sequenced as part of a GBM genome sequencing project [3] and has the next most common alteration in the RTK/AKT pathway: an inactivating PTEN mutation.

To investigate the active cell signaling pathways in GBM stem-like cells, 020913 and 060919 cells were analyzed using the phospho-RTK array and phospho-kinase array. These arrays simultaneously determine relative phosphorylation levels in over 40 different kinases.

**Figure 1:**
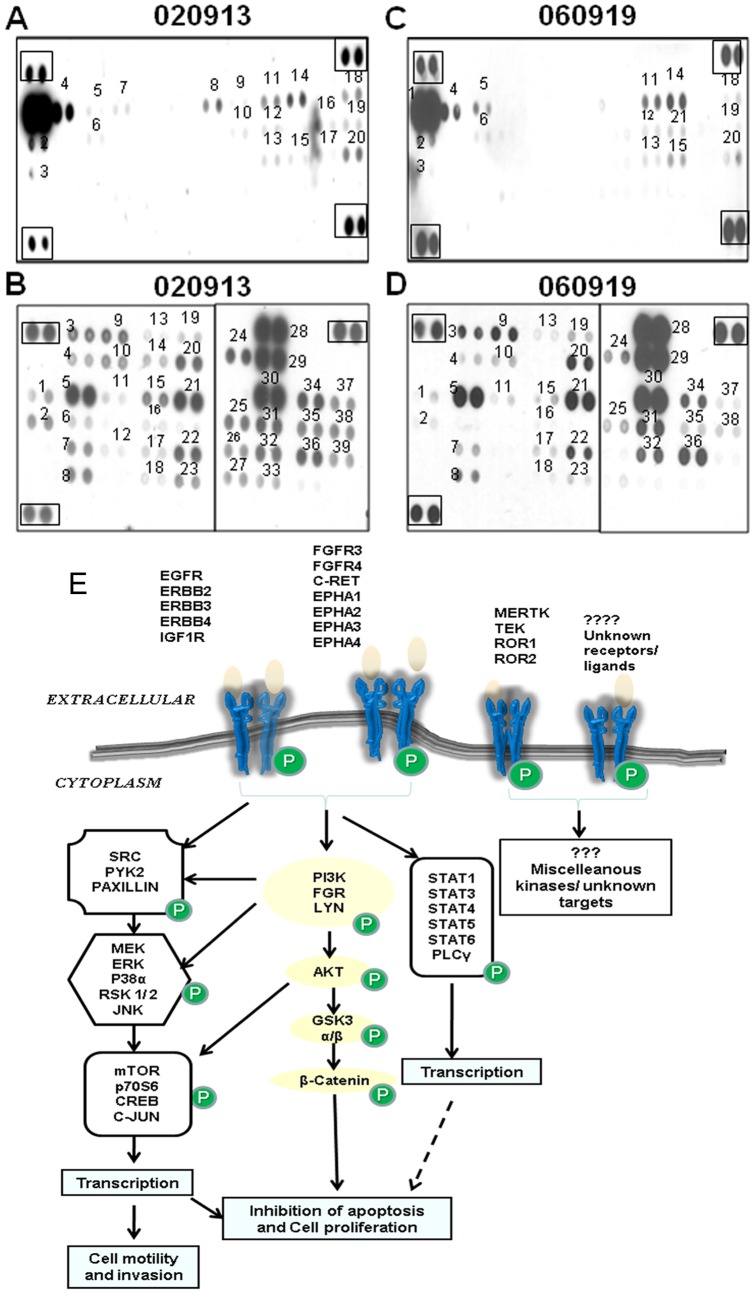
Kinase array analysis demonstrates high levels of phosphorylated EGFR, ERBB2, CREB and p53. 020913 and 060919 GBM oncospheres demonstrated activation of multiple receptor tyrosine kinases (A and C), and non-receptor tyrosine kinases (B and D). The RTKs that were phosphorylated (A and C) are numbered as follows: 1. p-EGFR 2. p-MER 3. p-TIE2 4. p-ERBB2 5. p-ERBB3 6. p-MSP R 7. p-ERBB4 8. p-FGFR3 9. p-FGFR4 10. p-M-CSF R 11. p-Insulin R 12. p-c-RET 13. p-EPHA1 14. p-IGF-IR 15. p-EPHA2 16. p-ROR2 17. p-EPHA3 18. p-DLK 19.p-TIE1 20. p-EPHA4 21. p-ROR1. Similarly, non receptor tyrosine kinases (B and D) that were phosphorylated are numbered as follows: 1. p-TOR (S2448) 2. p-SRC (Y419) 3. p-p38α (T180/Y182) 4. p-MEK1/2 (S218/S222, S222/S226) 5. p-CREB (S133) 6. p- LYN (Y397) 7. p-YES (Y426) 8. p-CHK-2 (T68) 9. p-ERK1/2 (T202/Y204, T185/Y187) 10. p-MSK1/2 (S376/S360) 11. p-HSP27 (S78/S82) 12. p-FGR (Y412) 13. p-JNK pan (T183/Y185, T221/Y223) 14. p-AMPKα1 (T174) 15. p-AMPKα2 (T172) 16. p-STAT2 (Y689) 17. p-STAT3 (Y705) 18. p-STAT6 (Y641) 19. p-GSK-3α/β (S21/S9) 20. p-AKT (Y473) 21. p-β-CATENIN 22. p- STAT5b (Y699) 23. p-STAT5a/b (Y699) 24. p- Akt (T308) 25. p-p70 S6 kinase (T421/S424) 26. p-p70 S6 kinase (T229) 27. p-STAT1 (Y701) 28. p-p53 (S392) 29. p-p53 (S46) 30. p-p53 (S15) 31. p-RSK1/2/3 (S380) 32. p-RSK1/2 (S221) 33. p-STAT4 (Y693) 34. p-p27 (T157) 35. p-c-JUN (S63) 36. p-eNOS (S1177) 37. p-PAXILLIN (Y118) 38. p-PLCγ (Y783) 39. p-PYK2 (Y402). Figure 1E: Schematic representation of activation of multiple tyrosine kinases in GBM oncosphere lines. Only the kinases with a known role in cell proliferation and/or transcription have been depicted.

Analysis of the subsequent phosphorylation profiles revealed that both the GBM oncosphere cell lines were associated with extensive activation of multiple tyrosine kinases including both receptor and non-receptor tyrosine kinases as shown their phosphorylation status (Figure 1A and 1B). The co-activated RTKs identified were p-EGFR, p-ERBB2, p-ERBB3, p-ERBB4, p-FGFR3, p-FGFR4, p-INSULIN R, p-c-RET, p-IGF-IR, p-EPHA2, p-MSP R, p-ROR1, p-ROR2, p-M-CSF R, p-EPHA3, p-DLK, p-TIE1, p-EPHA4 and p-EPHA1. Investigation of the phosphorylation status of the cytoplasmic non-receptor tyrosine kinases revealed that pathways including AKT, MAPK, JAK-STAT, Wnt/β-catenin, PKA (CREB), PLCγ (PKC) signaling were active in GBM oncosphere cells.

These results indicate that multiple kinases are activated in GBM oncosphere cells and have been summarized in Figure 1E. These results suggest that targeting multiple tyrosine kinases might be more effective in GBM cells.

### Kinase Inhibitors in Vitro Work Best in Combination

Eleven different RTK inhibitors were evaluated for their ability to inhibit GBM oncosphere growth. The IC_50_ values of these eleven inhibitors were evaluated using an alamarBlue based assay and are listed in Table S1.

To initially test our hypothesis of combined inhibition, the various pair-wise combinations of RTK inhibitors were tested for growth suppression at 25% (one fourth) and 10% (one tenth – Figure S2) of their respective IC_50_ concentrations. Thirty-two such combinations were evaluated and showed that single agents, did not substantially alter growth, and only certain combinations suppressed growth (Figure S3 and Figure S4). The extent of growth fold inhibition was calculated by dividing the alamarBlue fluorescence values for the treated cells with fluorescence values for cells treated with the vehicle.

**Figure 2:**
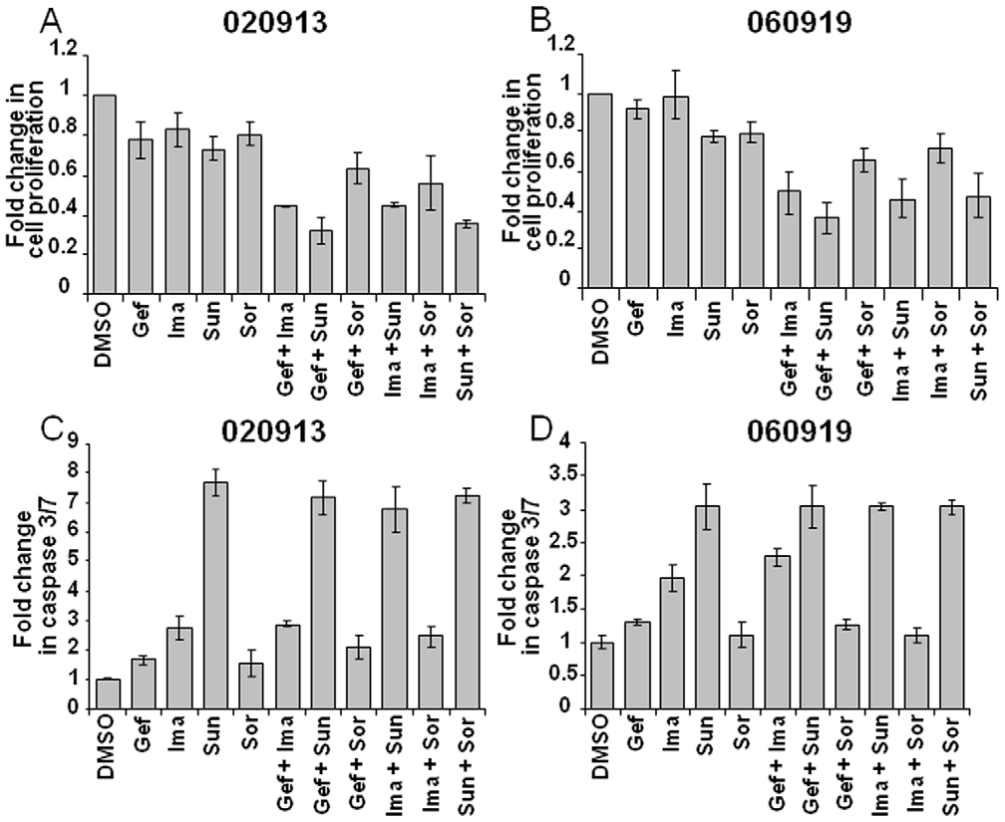
Drug combinations consisting of multi tyrosine kinase inhibitor sunitinib works best in reducing growth and inducing apoptosis in GBM oncospheres. Fold change in proliferation is shown for 020913 (A) and 060919 (B) cells when treated with FDA-approved RTK inhibitors at 25% of their IC_50_ concentration. Combination of gefitinib (5 µM) and sunitinib (10 µM) demonstrate synergism in inhibiting cell growth. Other combinations of gefitinib and imatinib (15 µM), sunitinib and sorafenib (1 µM), and imatinib and sunitinib also showed increased growth inhibition of GBM cells. C and D: Caspase 3/7 assay demonstrating that sunitinib alone induces caspase 3/7 expression in 020913 cells (C) and 060919 cells (D), whereas treatment with Gefitinib, Imatinib and Sorafenib did not show caspase 3/7 release. Also, combinations containing sunitinib did not demonstrate an increased caspase release.

With the aim of translating the drug combinations to possible human use, we eventually focused only on drugs currently approved by the FDA that targeted the RTKs mutated in GBM. Erlotinib did not show significant inhibition even at a concentration of 100 μM. Lack of erlotinib activity may be attributed to its low solubility in DMSO compared to gefitinib and was therefore eliminated from the subsequent analysis. Remaining were these four inhibitors: gefitinib, imatinib, sunitinib and sorafenib. To test for synergistic cytotoxic effect on GBM oncospheres, one tenth (Figure S2) and one fourth the IC_50_ values were next used. Single drugs and pair-wise combinations of these drugs were analyzed in GBM oncosphere lines for proliferation and caspase induction. Drug combinations containing sunitinib were best at inducing apoptosis (Figure 2C and 2D), and the best combination for inhibiting growth appeared to be gefitinib plus sunitinib (Figure 2A and 2B).

### Gefitinib Plus Sunitinib Combination Blocks Regrowth of GBM Oncospheres

**Figure 3:**
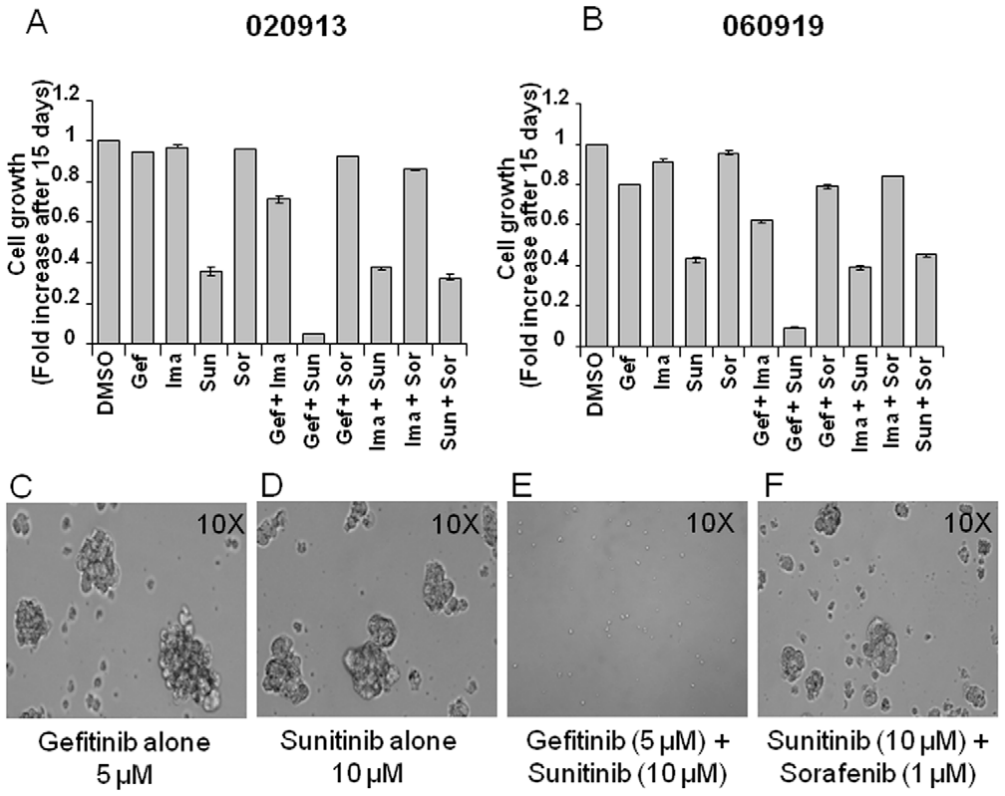
Oncospheres treated with sunitinib and gefitinib show no regrowth after treatment. 020913 cells (A) and 060919 cells (B) were treated for 24 hours with RTK inhibitors as single agents or in combination at 25% of their IC_50_ concentration. The drug was withdrawn after 24 hours and the cells were allowed to grow for 2 weeks. Treated cells were analyzed for their growth kinetics using alamar blue assay. GBM cells treated with the RTK combination consisting of gefitinib and sunitinib were unable to re-grow, whereas GBM cells treated with other drugs or combinations survived and re-grew. Analysis of neurosphere formation ability demonstrated that 020913 cells treated with gefitinib (C) or sunitinib (D) or other combinations like sunitinib and sorafenib (F) could form neurospheres after withdrawal of the drug, whereas cells treated with gefitinib and sunitinib (E) could not form any neurospheres.

To investigate the differences observed in the growth inhibition and caspase assay of the GBM oncospheres, the ability of the oncospheres to recover and proliferate following treatment with RTK inhibitors was analyzed. Cells were treated with RTK inhibitors at 25% of IC_50_ concentrations for 24 hours. The drugs were withdrawn after 24 hours and the ability of the oncospheres to regrow was assessed after two additional weeks of culture in the growth media using alamar blue cell growth assay. The growth assessment revealed that oncospheres treated with single agents or with the combination of RTK inhibitors were able to regrow with the exception of the cells treated with the combination of gefitinib and sunitinib (Figure 3). Moreover, observation of the cells treated with the drugs with light microscopy revealed that cells treated either with single agents or combinations of RTK inhibitors other than gefitinib and sunitinib were able to form oncospheres, whereas the cells treated with a combination of gefitinib and sunitinib were unable to form oncospheres. 020913 cells formed fewer neurospheres when treated with sunitinib compared to gefitinib reflecting the growth inhibition seen earlier in alamar blue assay. This observation suggests that gefitinib and sunitinib forms a specific combination that effectively inhibits growth of GBM oncospheres.

### Gefitinib Sunitinib Combination Inhibits Kinase Activity in GBM Oncosphere Cells

**Figure 4:**
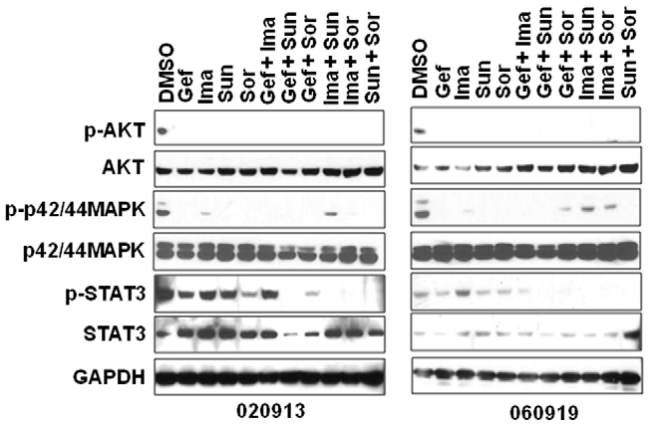
Kinase inhibition after treatment with RTK inhibitors. A. Combining receptor tyrosine kinase inhibitors suppress downstream effectors of growth factor signaling. p-STAT3 was blocked only by the combination treatment, whereas p-AKT was blocked by all the drugs either as single agents or in combinations. The combinations of gefitinib and sunitinib as well as sunitinib and sorafenib were able to inhibit p-AKT, p-MAPK and p-STAT3 in both the GBM oncosphere lines.

The 020913 and 060919 GBM oncosphere lines were treated for 24 hours with the RTK inhibitors either as single agents or in combination, to determine downstream changes in cancer-related signaling transduction. The cell lysates were analyzed for phosphorylation status of three major cell signaling pathways including AKT, MAPK and STAT3 (Figure 4). All the RTK inhibitors either as single agents or in combinations were able to block phosphorylation of AKT. Some drugs could only inhibit phosphorylation of either STAT3 or MAPK, but not both. The combination of gefitinib and sunitinib, and sunitinib and sorafenib were the only ones able to simultaneously block the phosphorylation of AKT, MAPK and STAT3 in both of the GBM oncosphere lines. The inhibition of p-AKT, p-MAPK and p-STAT3 by gefitinib and sunitinib as well as sunitinib and sorafenib correlated with the ability of these combinations to effectively inhibit GBM oncosphere growth, although this does not directly demonstrate mechanism.

To detect cell signaling changes in response to the gefitinib and sunitinib combination, 020913 oncospheres were treated for six hours and lysates analyzed with the phospho-specific kinase antibody array. The treatment decreased phosphorylation in EGFR, ERBB2, FGFR3, MER, TIE2, INSULIN R, C-RET, EPHA1, DLK, TIE1, EPHA4, AKT, MAPK, PKA (CREB), JAK-STAT, SRC, c-JUN and p53. There was an observed increase in phosphorylation of IGF-IR and EphA2 (Figure S5A and S5B).

### RTK combination consisting of gefitinib and sunitinib demonstrate limited efficacy in vivo

**Figure 5:**
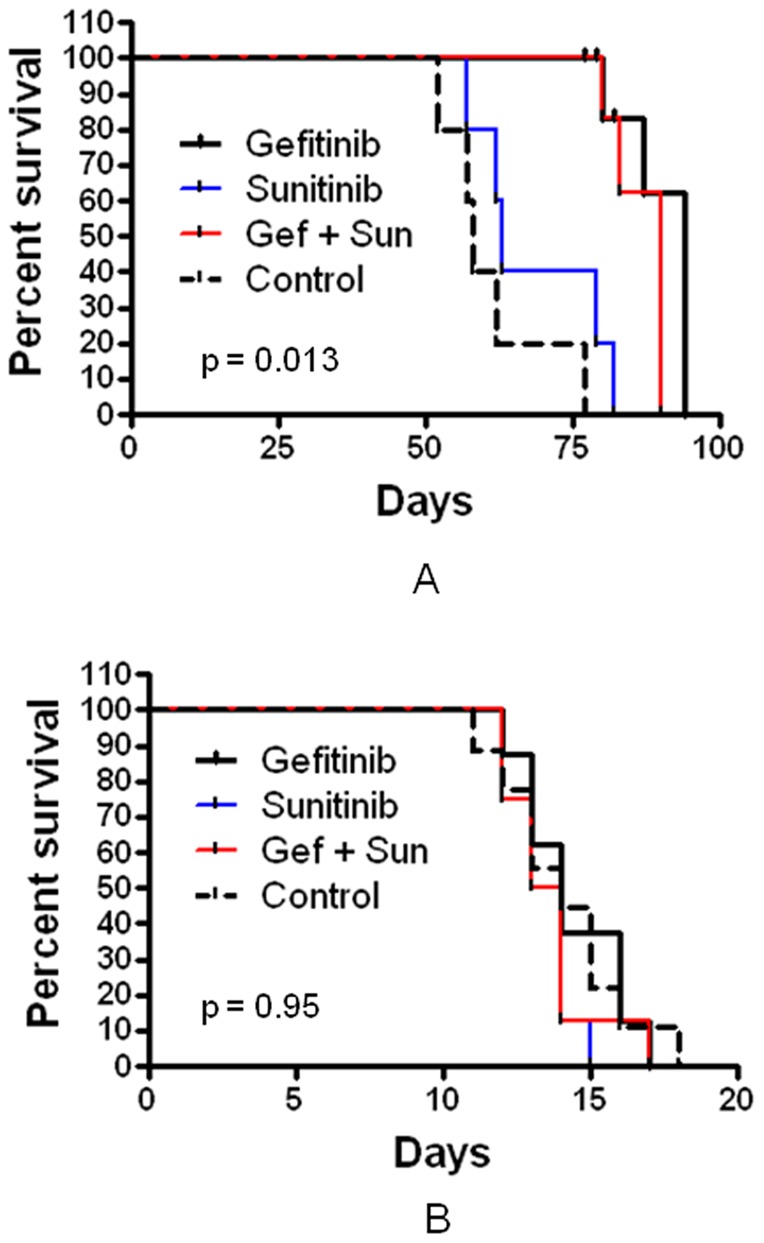
A: Mice implanted intracranially with 020913 GBM oncosphere cells were treated with gefitinib (75 mg/kg), sunitinib (15 mg/kg) and a combination of gefitinib (75 mg/kg) and sunitinib (15 mg/kg ). Gefitinib alone could significantly (p = 0.0001) improve survival in the animals compared to control animals. Sunitinib did not show any efficacy either when used alone (p = 0.13, compared to control) or when combined with gefitinib (p = 0.18, compared to gefitinib alone). Figure 5B: Rats were implanted intracranially with 9L tumor 1 mm^3^ tumor pieces. Rats were then treated with gefitinib (50 mg/kg), sunitinib (8 mg/kg) and a combination of gefitinib (50 mg/kg) and sunitinib (8 mg/kg). None of the drugs including the combination of gefitinib and sunitinib showed any efficacy (p = 0.9).

The efficacy of best *in vitro* RTK combination consisting of gefitinib and sunitinib was evaluated *in vivo* in an intracranial glioblastoma xenograft model. Five hundred thousand 020913 GBM stem cells were implanted intracranially in each of the twenty athymic nude mice. The mice were divided into four groups of five each. The treatment groups consisted of mice treated with gefitinib alone, sunitinib alone and combination of gefitinib and sunitinib. The control group consisted of five mice gavaged with phosphate buffered saline (PBS). In order to mimic the human trial, the doses of the drugs administered in animals were equivalent to the FDA approved human doses (dose calculation in Data S1). The mice in the treatment group were treated with gefitinib alone (75 mg/kg), sunitinib (15 mg/kg) alone and combination of gefitinib (75 mg/kg) and sunitinib (15 mg/kg) three days a week. The median survival of the mice from the control group was 58 days, whereas the mice receiving the RTK inhibitors gefitinib and the RTK combination demonstrated a significantly improved survival (p = 0.0001) with median survival of 94 and 90 days respectively (Figure 5A). Mice treated with sunitnib alone had a median survival of 63 days and was not significantly different from the control group (p = 0.13). The results also demonstrate that addition of sunitinib to gefitinib had no significant impact on median survival of mice treated with gefitinib alone (p = 0.18).

The *in vivo* study was also repeated in a different genetic background using a rat syngeneic gliosarcoma model. 9L gliosarcoma 1 mm^3^ tumor pieces were implanted intracranially in 32 Fisher 344 rats. The rats were divided into four groups of 8 each and were gavaged with gefitinib alone, sunitinib alone, gefitinib and sunitinib combination and PBS. The animals were treated every Monday, Wednesday and Friday as described earlier and the doses were adjusted for the rats as described earlier. Surprisingly, in this study none of the drugs including gefitinib and the gefitinib and sunitib combination showed any efficacy (Figure 5B). These results demonstrate that the outcome of drug efficacy testing in animal models is very much influenced by the underlying genetic backbone of the tumor cell line, and very likely as well the ability for drugs to reach intracranial tumors.

## Discussion

The goal of this work was to determine if we could find a combination of approved RTK inhibitors that might be superior to single agent therapy, and test this combination in preclinical animal models of glioblastoma. Monotherapy of RTK targeting agents have been largely ineffective and there is enough *in vitro* experimental evidence to support the use of combination therapy targeting multiple tyrosine kinases [7], [8], [9], [10], [11].

We first identified possible effective RTK combination using *in vitro* cell proliferation studies. Next we planned to test efficacy in improved preclinical animal models at FDA approved doses to try and mimic what might be achievable in a clinical trial.

In this study, gefitinib and sunitinib was the best *in vitro* combination, based on its ability to reduce proliferation and kill GBM oncospheres. The pattern of effective inhibitor combinations suggests that successful simultaneous inhibition of EGFR and PDGFR and other tyrosine kinases was necessary.

In spite of the *in vitro* prediction, our results *in vivo* differed quite significantly. We did achieve a survival benefit in animals, but evidence indicated this was only for the gefitinib, and only in the cell line with EGFR amplification, where there is existing data to suggest that a single EGFR inhibitor might have a modest survival benefit in those tumors most dependent on EGFR signaling [12].

The results of the *in vivo* efficacy studies demonstrate that gefitinib alone could improve survival in 020913 GBM xenograft models by 62% compared to untreated controls, whereas the same drug was a completely ineffective when tested at similar concentrations in a syngeneic 9L rat gliosarcoma model. The differences in the results could be attributed to the genetic makeup of the cells. 020913 cells are human GBM derived neurosphere line that has always been propagated in a serum free media supplemented with EGF and FGF [13]. It is possible that the cell culture conditions would select the cells that are more dependent on EGF and FGF for their growth. Moreover, 020913 cells have EGFR amplification and therefore these cells would be more responsive towards EGFR inhibitors such as gefitinib. On the contrary, 9L cells are grown in serum containing medium and have no specific dependence on EGF for growth and may not be inhibited by mere EGFR inhibition.

Sunitinib was the only RTK inhibitor that induced apoptosis in GBM oncosphere cells (Figure 3), whereas all the other RTK inhibitors were cytostatic. However, sunitinib failed to demonstrate any efficacy in our preclinical GBM animal models affirms the recently published phase II clinical trial data demonstrating limited efficacy of sunitinib in GBM patients [14]. The most likely explanation for this is that sunitinib cannot reach effective intracranial tumor concentrations at these doses. Sunitinib failed to work *in vivo* in 9L cells that are significantly more sensitive to sunitinib *in vitro* (9L cells have at least 5–10 times lower IC_50_ for sunitinib compared to GBM oncosphere lines, Table S1).

Our observations are consistent with a previous report demonstrating responsiveness of GBM patients co-expressing EGFRvIII and PTEN to EGFR inhibitors [12]. Guillamo et al. demonstrated that loss of PTEN makes GBM xenografts resistant to gefitinib in an ex vivo brain slice model [15]. Similarly, EGFR copy number has been shown to be a predictor of gefitinib related survival benefit in advanced non small cell lung cancer patients [16]. It is also likely that other EGFR mutations, such as point mutations within EGFR or PIK3CA or PTEN and the activation of PI3K/AKT pathway need to be considered when considering response to EGFR inhibitors [17].

Clinical trial evaluating erlotinib, another EGFR inhibitor had insufficient activity in GBM patients and no clear biomarker could be identified that was associated with a response to erlotinib [18]. However, a study by Sarkaria et al. [19] identified two GBM xenografts that were sensitive to erlotinib. Sarkaria et al. concluded that amplification or mutation of EGFR and presence of wild type PTEN was required but not enough for ensuring sensitivity to erlotinib. One other key difference between our study and the one by Sarkaria et al. and Van den Bent et al. is the choice of inhibitors. Although, both gefitinib and erlotinib target EGFR, they are different small molecules and have a differing pharmacokinetic profile. Gefitinib can cross blood brain barrier effectively and dephosphorylate EGFR [20], whereas erlotinib is a substrate for p-glycoprotein and breast cancer resistance protein and therefore has a limited brain penetration [21].

Immunoblotting analysis of 020913 cells when treated with different RTK inhibitors *in vitro* suggests that to achieve complete growth inhibition of GBM oncosphere lines all of the three pathways involving AKT, STAT3 and MAPK that are downstream of RTKs should be inhibited. Activation of any one of these pathways may enable the oncosphere cells to develop a resistance mechanism and regrow- or simply continue growth. Gefitinib or sunitinib or the other RTK inhibitors when used as single agents could only inhibit p-AKT and not p-STAT3 (Figure 4) and the cells could regain their growth potential immediately after the drug was withdrawn (Figure 3). These observations suggests that while devising a rational combination therapy for treating GBMs, a multipronged approach should be used that is effective enough to shut down all three oncogenic signaling pathways *in vivo*. This suggests a real challenge for targeted therapy of GBM where very few drugs can reach effective intra-tumor concentrations. Despite the difficulties of developing drugs for direct delivery either by polymer wafer or convention enhanced delivery; these may have to be seriously considered if combination targeted therapy with the present available inhibitors is to achieve survival benefit in clinical trials.

Our results are consistent with previous evidence that EGFR inhibition might be beneficial in a subset of patients [12], [19], but we favor a combination approach based on our *in vitro* results. This conclusion is based on the fact that only combined inhibition was best at inhibiting signaling in all key pathways. Clinical trials with EGFR inhibitors in GBM have had only modest benefit at best even when accounting for EGFR pathway biomarkers [18]. Recently mathematical modeling of EGFR inhibition in metastatic colon cancer suggests that the likely number of preexisting resistance mutations make it virtually impossible for a single targeting agent to prevent tumor re-growth [22]. Overall, effort might be better used to identify better combinations.

Although our best *in vitro* combination failed to improve over single agent *in vivo*, the results are informative. We conclude that a combination could include gefitinib, in particular if can be used at higher doses than used currently in the clinic. A kinase inhibitor targeting PDGFRA and/or FGFR or other frequently activated tyrosine kinases that can reach effective intra-tumor concentrations before dose limiting toxicity is a likely candidate for a combination with an EGFR inhibitor for a potentially more effective therapy. Enhanced delivery systems to intracranial tumors may be necessary as part of a successful strategy.

## Materials and Methods

### GBM Stem-Like Cell Lines and Serum Grown Cell Lines

GBM oncosphere line 020913 was from Sara Piccirillo and Angelo Vescovi, Università degli Studi Bicocca-Millan, Italy. GBM oncosphere lines 060919 and 020913 were cultured in stem/progenitor cell media (Cambrex, East Rutherford, NJ) containing EGF (20 ng/ml) and FGF (10 ng/ml) (PeproTech Inc., Rocky Hill, NJ) and maintained at 37°C in a humidified incubator with an atmosphere of 5% CO_2_. 020913 cells harbor EGFR amplification (Figure S1). 060919 cells were generated at Johns Hopkins and were profiled by whole genome sequencing as a part of glioblastoma genome project (sample # BR23X) [3]. Human U87 glioblastoma and rat 9L glioma cells were maintained in DMEM supplemented with 10% FBS and Penicillin/ streptomycin at 37°C with 5% CO2.

### Tyrosine Kinase Inhibitors

Gefitinib (Iressa™) and erlotinib (Tarceva™) were kindly provided by Dr. Nisana Namwat (Khon Kaen University, Khon Kaen, Thailand). Imatinib mesylate (Gleevec™) was synthesized by American Custom Chemicals Corporation (San Diego, CA) and supplied by the Ludwig Institute for Cancer Research. Sunitinib malate (Sutent™) and sorafenib *p*-toluenesulfonate (Nexavar™) were purchased from LC laboratories (Woburn, MA). MET inhibitor (SU11274), FGFR inhibitors (SU4984, SU5402 and PD173074) and PDGFR tyrosine kinase inhibitor III were purchased from Calbiochem (La Jolla, CA). Inhibitors were dissolved in dimethylsulfoxide (DMSO) at a stock concentration of 10 mM or 100 mM and stored at −20°C until used.

### Phospho-kinase Antibody Array and Immunoblotting

Profiling of receptor tyrosine kinases (RTKs), kinases and their protein substrates phosphorylation were analyzed by using Human Phospho-RTK Array Kit (#ARY-001, R&D systems, Minneapolis, MN) and Human Phospho-Kinase Array Kit (#ARY-003, R&D systems, Minneapolis, MN) according the manufacturer's instructions using protein extracts from GBM stem-like cell lines (020913 and 060919). Cell lysates were diluted and incubated overnight with the array membrane. The array was washed to remove unbound protein, incubated with an antibody cocktail, and then developed using streptavidin-horseradish peroxidase and chemiluminescent detection reagents.

Cells (2×10^5^) were seeded in 6 well plates (Becton Dickinson, Franklin Lakes, NJ). After overnight incubation, cells were treated with single drugs and combinations, and then harvested at 24 hours time point. Protein lysates were prepared using RIPA buffer and immunoblot analysis was performed as previously described [23].

### Cell Proliferation and Apoptosis Assays

GBM stem-like cell proliferation was assessed using an alamarBlue® assay (Invitrogen, Carlsbad, CA). GBM oncospheres of the appropriate size range were plated (5×10^2^) in black clear-bottom 96 well plates (Becton Dickinson) and incubated overnight. The following day, drugs were added as single agents or in combination at designated concentrations and then 20 μl of 10X alamarBlue® was added. The volume in each well was made up to 200 μl with the growth medium. After 72 hours incubation, fluorescence was measured on a Perkin Elmer Wallac 1420 Multilabel counter (Perkin Elmer, Turku, Finland) with a 540 nm excitation filter and a 590 nm emission filter. Experiments were done twice with 6 replicates for each experiment. For the IC50 calculation, GBM oncosphere cells were treated with RTK inhibitors at nine different concentrations ranging from 100 μM to 1 nM (100 μM, 50 μM, 10 μM, 5 μM, 1 μM, 0.5 μM, 0.1 μM, 10 nM and 1 nM) and DMSO was used as a vehicle control. The DMSO volume was kept uniform at 1% of the total volume. After reading the alamarBlue fluorescence at 72 hours, Graphpad prism 5 was used to calculate the IC50 values. Apoptosis assays were performed using the Caspase-Glo® 3/7 Assay (Promega Corporation, Madison, WI) according to manufacturer's instructions and as described earlier [23].

### In Vivo GBM Oncosphere Xenograft Model and 9L Gliosarcoma Syngeneic Model

All the animal studies were approved by the Johns Hopkins Animal Care and Use Committee. Twenty female athymic nude mice were anesthetized with a mixture of ketamine and xylazine by intraperitoneal injection. After each animal was fully anesthetized, a small incision in the skin over the cranium was made. Using a surgical drill, a hole was made 1 mm lateral of midline and 1 mm lateral of Bregma over the parietal lobe. After drilling the hole, the animal was placed in a stereotactic frame and 500,000 020913 cells were implanted at a depth of 2.5 mm using a Hamilton syringe. After implantation, the incision was closed with surgical staples.

The 9L gliosarcoma was obtained from Marvin Barker, MD, (University of California, San Francisco, Brain Tumor Research Center, San Francisco, CA). For tumor piece implantation, 9L tumor pieces measuring 2 mm^3^ were passaged in the flank of F344 rats (female, 150–200 g) every 3 to 4 weeks. For intracranial implantation, the 9L gliosarcoma tumor was surgically excised from the carrier animal, cut into 1-mm^3^ pieces, and placed in sterile 0.9% saline on ice. The excised tumor was then intracranially implanted in 32 F344 female rats weighing 150–200 gms as described previously [24].

### Animal Drug Treatment

All the animals were treated with drug doses equivalent to FDA approved human doses. A weekly human dose was calculated for gefitinib and sunitinib and was converted to a mouse or a rat dose using the formula prescribed by Reagan-Shaw et al [25]. The weekly animal dose was then equally divided into three installments to be delivered on Monday, Wednesday and Friday and the treatment continued until the animals were dead or sacked due to tumor burden.

### Statistical Analysis

Statistical and graphical analyses were performed using GraphPad Prism 5 (GraphPad, LaJolla, CA). The Student T-test was used to compare the growth inhibition in various groups. Animal survival was analyzed using Kaplan-Meier survival and log rank test.

## Supporting Information

Figure S1 **Illumina analyses demonstrating amplification of EGFR in 020913 cells.**
(TIF)

Figure S2 **Fold change in proliferation of 020913 (A) and 060919 (B) cells.** 020913 and 060919 cells were treated with FDA-approved RTK inhibitors at 10% of their IC50 concentration. Combination of gefitinib (2 µM) and sunitinib (4 µM) demonstrate increased inhibition in 020913 and 060919 the cell growth compared to other combinations.
(TIF)

Figure S3 **RTK combination treatment.** 020913 cells were treated with FDA approved and unapproved RTK inhibitors at 25% of their IC50 concentrations.
(TIF)

Figure S4 **Vandetanib (Zactima™) combination treatment.** 020913 cells were treated with vandetanib in combination with other FDA approved drugs such as imatinib (ima), sunitinib (sun) and sorafenib (sor). Vandetanib is an EGFR and VEGFR inhibitor.
(TIF)

Figure S5 **Inhibition of phosphorylation in 020913 cells when treated with a combination of Gefitinib and Sunitinib**.
(TIF)

Table S1 **IC_50_ values of RTK inhibitors in GBM oncosphere and adherent cell lines.**
(DOCX)

Data S1 Calculation of FDA equivalent dose of RTK inhibitors for the animal studies.
(XLS)
