# Supplementary figures and images for: Evaluation of Tyrosine Kinase Inhibitor Combinations for Glioblastoma Therapy
Source: PLoS One. 2012 Oct 2;7(10):e44372. doi: 10.1371/journal.pone.0044372 (PMC3462750; doi:10.1371/journal.pone.0044372)

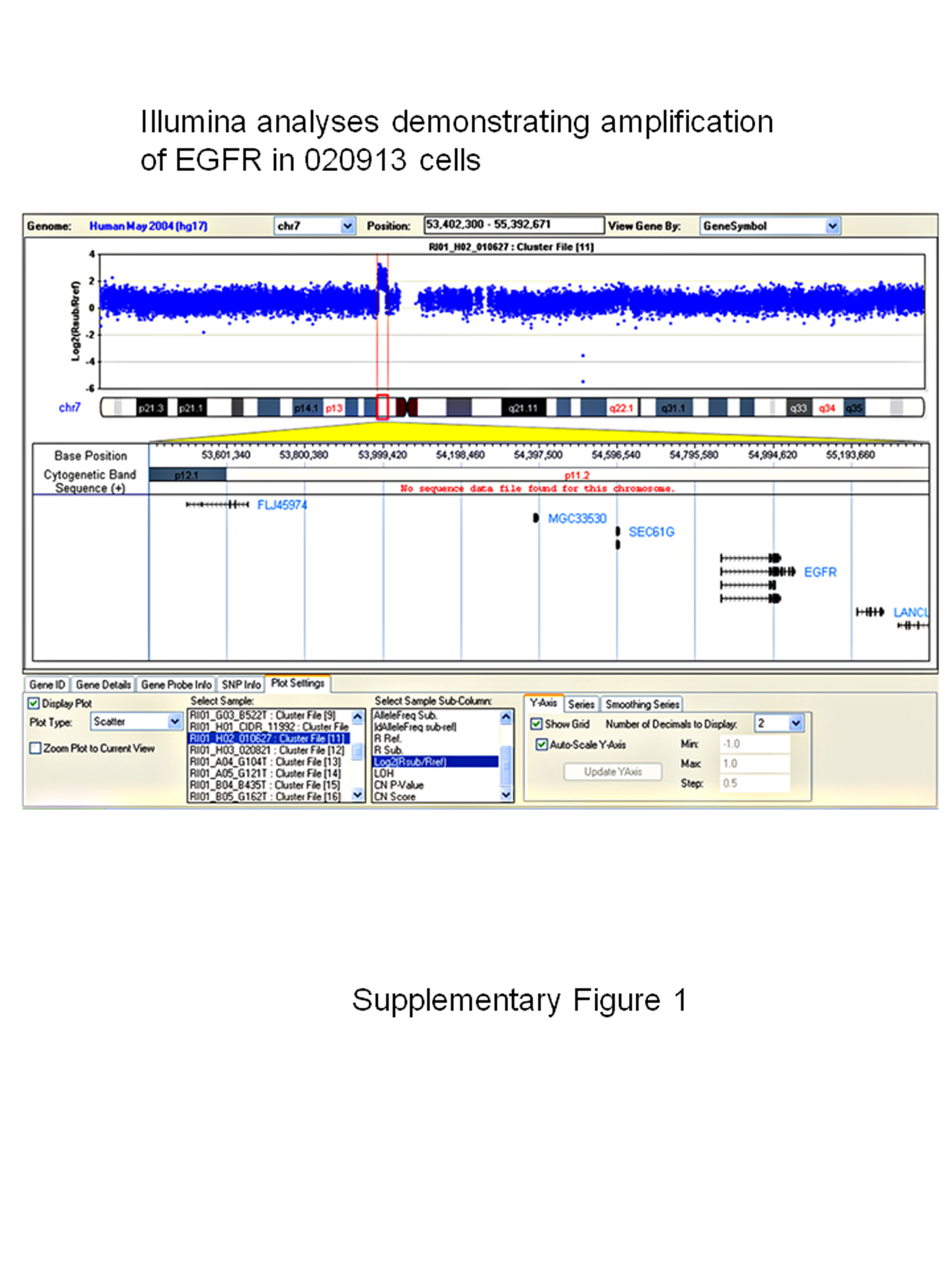

Supplement: Figure S1 — Illumina analyses demonstrating amplification of EGFR in 020913 cells. (TIF) [file pone.0044372.s001.tif]

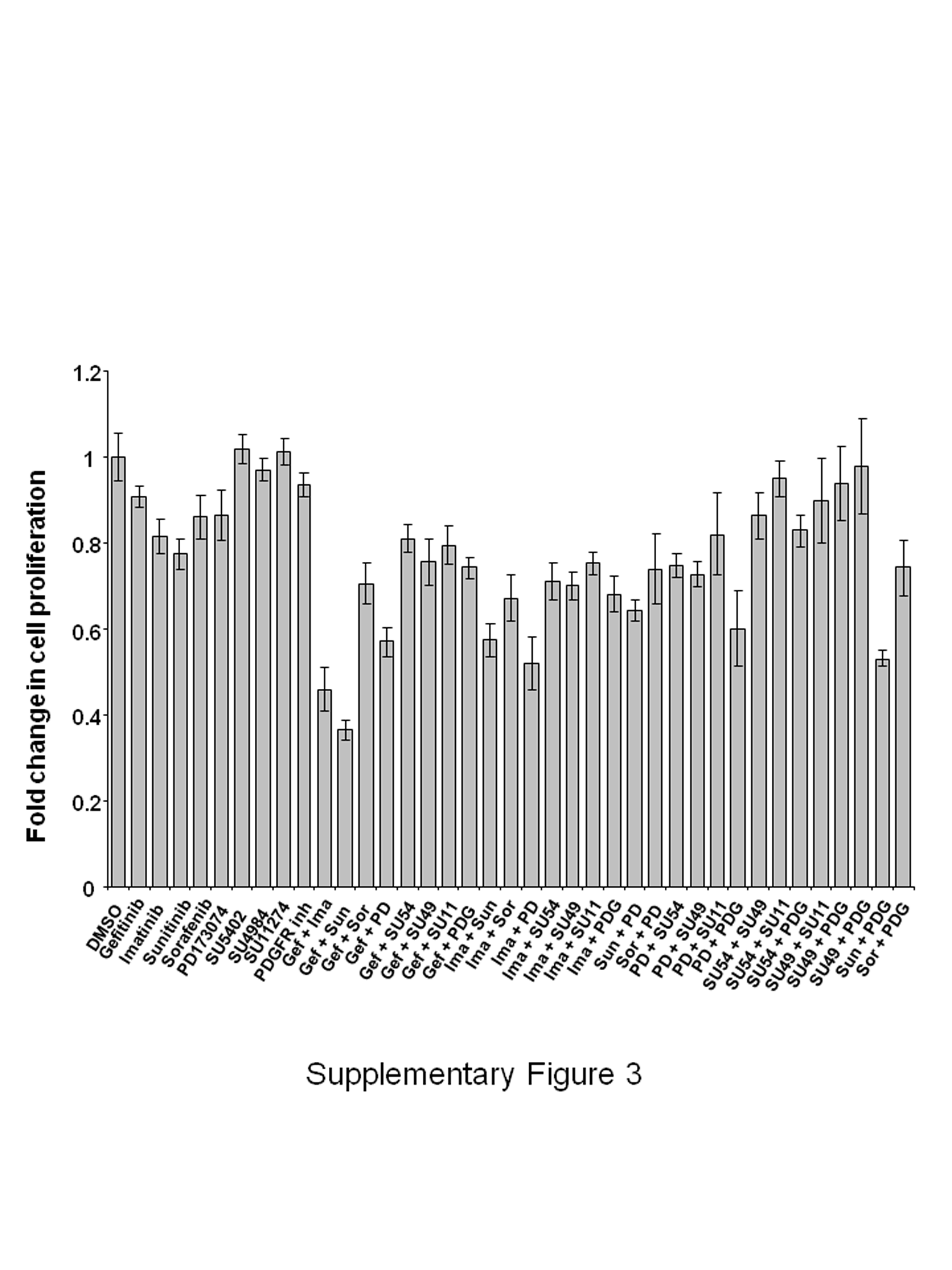

Supplement: Figure S3 — RTK combination treatment. 020913 cells were treated with FDA approved and unapproved RTK inhibitors at 25% of their IC50 concentrations. (TIF) [file pone.0044372.s003.tif]

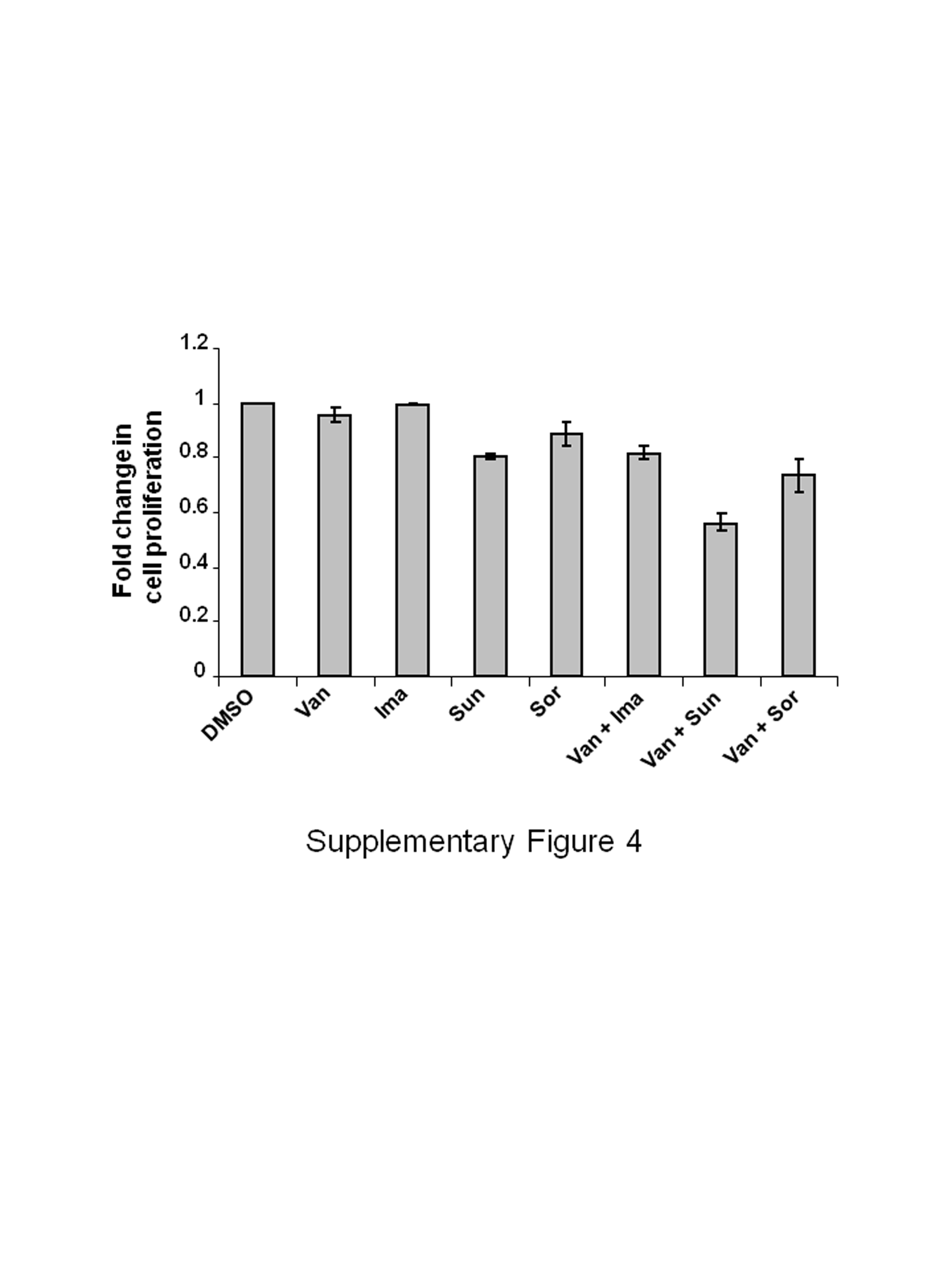

Supplement: Figure S4 — Vandetanib (Zactima™) combination treatment. 020913 cells were treated with vandetanib in combination with other FDA approved drugs such as imatinib (ima), sunitinib (sun) and sorafenib (sor). Vandetanib is an EGFR and VEGFR inhibitor. (TIF) [file pone.0044372.s004.tif]

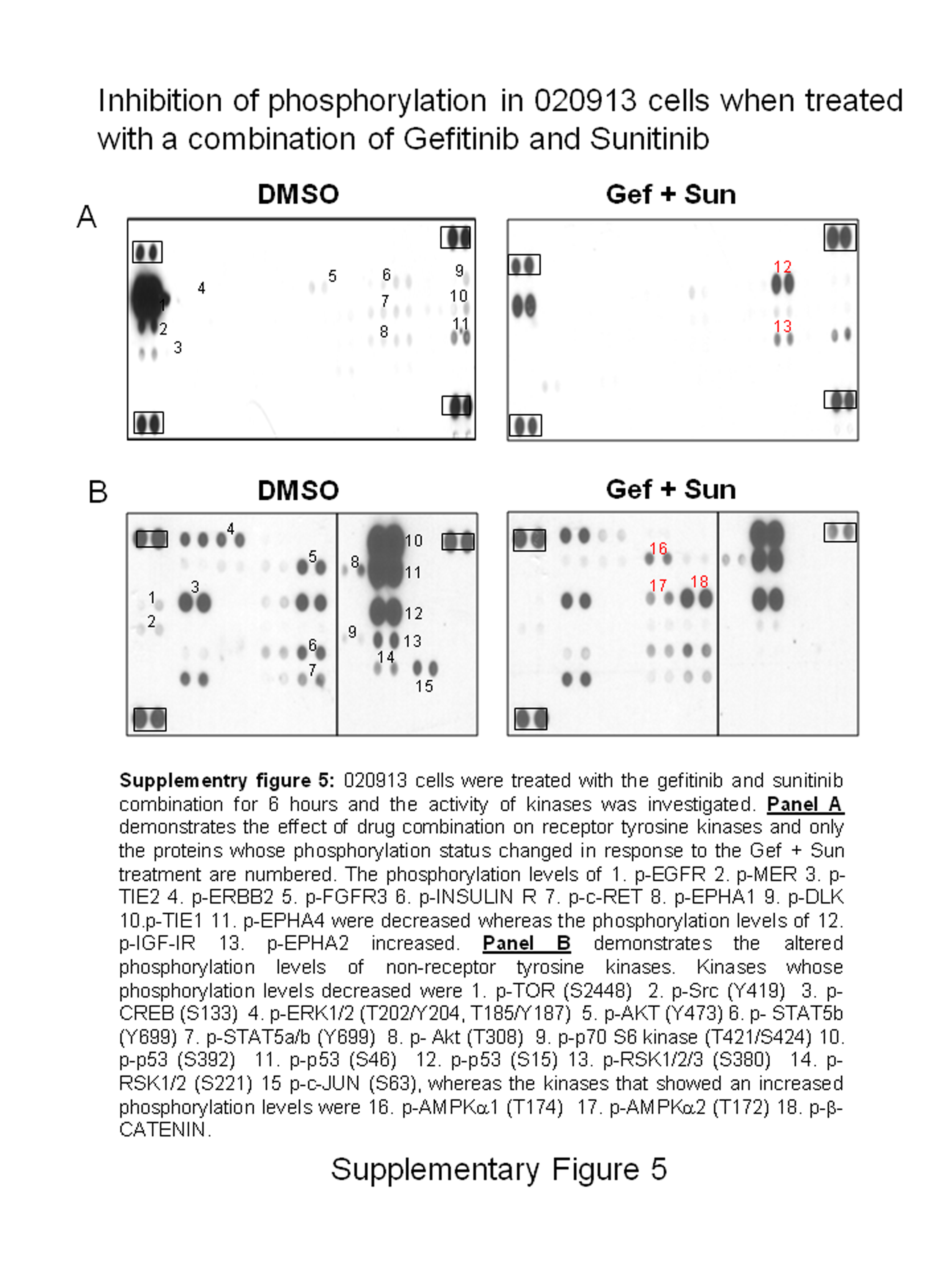

Supplement: Figure S5 — Inhibition of phosphorylation in 020913 cells when treated with a combination of Gefitinib and Sunitinib. (TIF) [file pone.0044372.s005.tif]
