# Supplementary material for: Evaluation of Tyrosine Kinase Inhibitor Combinations for Glioblastoma Therapy
Source: PLoS One. 2012 Oct 2;7(10):e44372. doi: 10.1371/journal.pone.0044372 (PMC3462750; doi:10.1371/journal.pone.0044372)
Supplement: Table S1 — IC50 values of RTK inhibitors in GBM oncosphere and adherent cell lines. (DOCX) [file pone.0044372.s006.docx]

**Supplementary Table 1:** IC_50_ values of RTK inhibitors in GBM oncosphere and adherent cell lines.

| **RTK inhibitors** | **Targets** | **IC50 (µM)** | | | | |
| --- | --- | --- | --- | --- | --- | --- |
|  |  | **020913** | **060919** | **U87** | **9L rat glioma** | **LN382** |
| Gefitinib | EGFR | 20.80 | 48.83 | 37.72 | 15.88 | 13.68 |
| Vandetanib | EGFR, VEGFR, RET | 16.92 | ND | 20.43 | ND | ND |
| Erlotinib | EGFR | >100 | ND | >100 | ND | ND |
| Imatinib | PDGFR, c-KIT, ABL | 64.33 | >100 | 76.46 | 40.68 | 76.40 |
| Sunitinib | PDGFR, VEGFR,  c-KIT, FLT3, CSF-1R, RET | 44.81 | 65.20 | 4.44 | 3.02 | 4.99 |
| Sorafenib | PDGFR, VEGFR,  c-KIT, c-Raf-1, b-Raf | 5.27 | 16.17 | 5.50 | 6.3 | 3.50 |
| PD173074 | FGFR, VEGFR | 29.12 | ND | 19.50 | ND | ND |
| SU5402 | FGFR | 55.81 | ND | >100 | ND | ND |
| SU4984 | FGFR | 12.96 | ND | >100 | ND | ND |
| SU11274 | c-MET | 2.36 | ND | 13.18 | ND | ND |
| PDGFR inhibitor | PDGFRA | 7.13 | ND | 10.32 | ND | ND |
